# Population Structure, Antimicrobial Resistance, and Virulence-Associated Genes in *Campylobacter jejuni* Isolated From Three Ecological Niches: Gastroenteritis Patients, Broilers, and Wild Birds

**DOI:** 10.3389/fmicb.2018.01676

**Published:** 2018-08-02

**Authors:** Yaidelys Iglesias-Torrens, Elisenda Miró, Pedro Guirado, Teresa Llovet, Carmen Muñoz, Marta Cerdà-Cuéllar, Cristina Madrid, Carlos Balsalobre, Ferran Navarro

**Affiliations:** ^1^Hospital de la Santa Creu i Sant Pau, Institut d’Investigació Biomèdica Sant Pau (IIB Sant Pau), Barcelona, Spain; ^2^Departament de Genètica i Microbiologia, Universitat Autònoma de Barcelona, Barcelona, Spain; ^3^Departament de Genètica, Microbiologia i Estadística, Universitat de Barcelona, Barcelona, Spain; ^4^Centre de Recerca en Sanitat Animal (CReSA)-IRTA, Campus de la Universitat Autònoma de Barcelona, Barcelona, Spain

**Keywords:** *Campylobacter jejuni*, PFGE, MLST, antimicrobial resistance, pathogenicity genes

## Abstract

*Campylobacter jejuni* is the causal agent of the food-borne infection with the highest incidence in Europe. Both poultry and wild birds are a major reservoir. To gain insight into the population structure, virulence potential, and antimicrobial resistance (AMR), a collection of 150 isolates from three different ecological niches (broilers, wild birds, and human patients) was studied. Despite the high genetic diversity found, the population structure defined two distinct clusters, one formed mostly by broiler and human isolates and another one by most wild bird isolates. The ST-21 complex exhibits highest prevalence (in humans and broilers), followed by ST-1275 complex (only in wild birds). The ST-48, -45, and -354 complexes were found in all three niches, but represent only 22 out of 150 studied strains. A higher occurrence of AMR and multidrug resistance was detected among broiler and human isolates. Moreover, significant differences were found in the distribution of certain putative virulence genes. Remarkably, many wild bird strains were negative for either *cdtA, cdtB*, or *cdtC* from the canonical strain 81-176, whereas all broiler and human strains were positive. These data suggest that the different variants of the *cdt* genes might be relevant for the efficient colonization of certain hosts by *C. jejuni*. Our study contributes to the understanding of the role of the diverse *Campylobacter* reservoirs in the transmission of campylobacteriosis to humans.

## Introduction

In the last decade, there has been an increase in the incidence of campylobacteriosis worldwide, which is especially significant in North America, Europe, and Australia (Cody et al., 2012; Harvala et al., 2016). This zoonosis is therefore of economic and public health concern (Havelaar et al., 2015). Particularly in the European Union, *Campylobacter* has outnumbered *Salmonella* as the most commonly reported cause of bacterial diarrheal disease in humans since 2005 (EFSA-ECDC, 2016). *Campylobacter jejuni* followed by *C. coli* are responsible for the vast majority of infections which lead to diarrhea and a range of other serious conditions affecting the gastrointestinal tract, especially in children under 5 years old (Eberle and Kiess, 2012; Kaakoush et al., 2015). Furthermore, two autoimmune neurological disorders, Guillain-Barre and Miller-Fisher syndromes have been associated in some patients with previous *C. jejuni* infections (Ang et al., 2001; van Doorn et al., 2008).

The natural reservoir of *Campylobacter* are a variety of domesticated and wild animals, including cats, dogs, cows, pigs, poultry, and wild birds, with the avian reservoir being the most important (Kaakoush et al., 2015). *Campylobacter* can be easily isolated from poultry retail meat, untreated water, unpasteurized milk, and soil (Workman et al., 2005; Engberg, 2006).

Since *C. jejuni* infections are usually self-limited and sporadic, antimicrobial therapy is only indicated in severe or long-lasting infections. The antimicrobials of choice are macrolides, sometimes combined with azithromycin and aminoglycosides in more severe cases of the disease (Moore et al., 2005; Bolinger and Kathariou, 2017). The use of fluoroquinolones and tetracyclines, often prescribed as empirical therapy for traveler’s diarrhea (Guerrant et al., 2001), has been reduced due to the dramatic increase of the frequency of resistant strains to these antimicrobial agents. Unfortunately macrolide resistance is increasingly being reported too (Lim et al., 2016).

Although the infection route has been described, the pathogenicity of *Campylobacter* remains mostly elusive. Very little is known on the bacterial factors exploited by *Campylobacter* during the infectious process. Besides the flagella and lipoligosaccharide, some putative virulence factors have been defined such as the cytolethal distending toxin (CdtABC), the fibronectin binding protein (CadF), and the invasion antigen (CiaB) (Young et al., 2007; Dasti et al., 2010). Moreover, factors that can be relevant for *Campylobacter* host adaptation and transmission have recently been identified by whole-genome sequencing studies (Pascoe et al., 2015; Thépault et al., 2017; Yahara et al., 2017).

Characterization of the population structure of *Campylobacter* strains recovered from different sources within a region and its antimicrobial resistance is critical to identify the major source of infection and to implement efficient control measures to reduce human exposure to the pathogen. Pulsed field gel electrophoresis (PFGE) and multilocus sequence typing (MLST) have been used to determine clonal diversity and population structure, respectively (Duarte et al., 2016). Moreover, gaining insight into the distribution of virulence-associated genes among strains might shed some light on the mechanisms exploited by *Campylobacter* to trigger infection.

In this study, we have characterized three populations of *Campylobacter* strains from different ecological niches: human patients suffering gastroenteritis, broilers, and wild birds. The relationship among the different strains in terms of population structure, antimicrobial resistance profile, and prevalence of virulence-associated genes has been established.

## Materials and Methods

### Bacterial Strains

A total of 150 isolates were obtained from feces of three different sources: human patients, broilers, and wild birds. Human isolates were obtained from the Santa Creu i Sant Pau Hospital (Barcelona) strain collection. Isolates were originally recovered from routine stool samples of 50 patients with diarrhea attended through the year 2014. All samples were anonymized. The patients were 54% children (≤ 16 years old), 30% adults, and 16% elderly people (≥ 65 years old). From these, 58% were men and 42% women. Domestic and wild avian isolates belong to a *Campylobacter* strain collection from previous studies conducted at IRTA-CReSA. Broiler (*Gallus gallus*) isolates were obtained from caecal samples collected in seven different slaughterhouses (A–G), from 2009 and 2011 to 2013. Broilers were originally from 24 farms located in Barcelona, Lleida, and Tarragona (Catalonia) (**Table 1**). Finally, the wild bird fecal samples from cloacal swabs were obtained during 2008–2013. Wild bird species sampled were: 1 northern shoveler (*Spatula clypeata*), 2 white storks (*Ciconia ciconia*), 5 common ravens (*Corvus corax*), 14 feral pigeons (*Columba livia*), 7 yellow-legged gulls (*Larus michahellis*), and 21 Audouin’s gulls (*Larus audouinii*) (**Table 2**). All wild bird samples were collected in Catalonia, except eight from Audouin’s gulls which were obtained from Alboran Island. Sampling methods were in compliance with the Ethical Principles in Animal Research of the Universitat Autònoma de Barcelona and the regulations required by the Ethics Commission in Animal Experimentation of the Generalitat de Catalunya. Field permits were authorized by Generalitat de Catalunya and Junta de Andalucia. All sampling procedures and/or experimental manipulations in the field were reviewed and approved as part of obtaining the field permit.

**Table 1:** Geographical relation between ST-complex and sequence types (STs) among *C*. *jejuni* from broiler caeca.

Slaughterhouse (*n*)	ST-complex^∗∗^	ST	Year	Farm	Farm Location	Strain
A (8)	S	441	2011	ES44	Lleida	B44
	ST-21	21	2013	ES28	Lleida	B36
	ST-45	45	2013	ES3	Lleida	B31
		137	2012	ES41	Lleida	B42
	ST-353	5	2013	ES52	Lleida	B29
	ST-354	354	2013	ES52	Lleida	B45
	ST-607	904	2011	ES28	Lleida	B47
			2013	ES3	Lleida	B38
B (6)	S	531	2013	ES138	Barcelona	B16
	ST-45	652	2013	ES138	Barcelona	B22
	ST-464	464	2013	ES129	Barcelona	B15
	ST-574	305	2015	ES129	Barcelona	B21
			2013	ES138	Barcelona	B25
	ST-607	1707	2013	ES129	Barcelona	B24
C (6)	S	7114	2013	ES191	Barcelona	B39
	ST-283	267	2013	ES191	Barcelona	B33
	ST-353	5	2013	ES193	Barcelona	B28
		400	2013	ES193	Barcelona	B18
	ST-48	48	2011	ES193	Barcelona	B09
	ST-464	464	2011	ES191	Barcelona	B08
D (9)	S	1710	2013	ES125	Tarragona	B26
	S	1710	2013	ES128	Tarragona	B35
	ST-21	50	2012	ES116/CAT5	Tarragona	B14/B50
		883	2013	ES116	Tarragona	B17
	ST-353	400	2013	ES116/ES115	Tarragona	B30/B40
	ST-354	8498	2013	ES125	Tarragona	B37
	ST-607	607	2013	ES128	Tarragona	B41
E (9)	S	1710	2011	ES84	Lleida	B10
	S	2331	2011	ES84	Lleida	B02
	ST-21	21	2013	ES84	Lleida	B20/B46
	ST-45	45	2013	ES85	Lleida	B27
	ST-257	2254	2012	ES202	Lleida	B13
	ST-353	356	2013	ES202	Lleida	B48
	ST-354	354	2013	ES84	Lleida	B23
	ST-607	7110	2012	CAT1	Lleida	B51
F (6)	ST-21	883	2011	ES156	Barcelona	B06
		50	2011	ES184	Tarragona	B07
	ST-206	46	2012	ES156	Barcelona	B49
	ST-353	400	2012	ES154	Lleida	B05
	ST-607	607	2011	ES147	Lleida	B04
		7110	2013	ES154	Lleida	B19
G^∗^ (6)	ST-257	367	2009	CT1/EU	Lleida	B57/B52, B53, B54, B55, B56


*n means number of strains. ^∗^Includes the six strains belonging to Clone 1 of PFGE pattern. ^∗∗^S means singleton.*

**Table 2:** Distribution of ST-complexes and sequence types (STs) of *C. jejuni* strains from wild birds.

Wild bird	ST-complex	ST	Location	Strains (Year of recollection)
Audouin’s gull (21)	ST-446 (1)	3552	Alboran Island	W12 (2010)
	ST-1034 (1)	4001	Ebro Delta	W05 (2009)
	ST-1275 (12)	1223	Ebro Delta	W03 (2009), W10 (2010), W15 (2010), W18 (2011), W21 (2011)
		1268	Ebro Delta	W22 (2011)
		1275	Ebro Delta	W04 (2009), W09 (2010), W20 (2010)
		1292	Alboran Island	W14 (2010), W16 (2010)
		3629	Ebro Delta	W19 (2010)
	Singletons (7)	1261	Ebro Delta	W11 (2010)
		1343	Alboran Island	W13 (2010), W23 (2010)
		2351	Ebro Delta	W17 (2011)
		4355	Alboran Island	W06 (2009), W07 (2009), W08 (2009)
Yellow-legged gull (7)	ST-45 (1)	45	Barcelona	W30 (2014)
	ST-48 (1)	48	Barcelona	W29 (2014)
	ST-354 (1)	354	Medes Islands	W28 (2013)
	ST-1275 (4)	637	Medes Islands	W24 (2012), W27 (2013)
		3049	Medes Islands	W26 (2013)
		8511	Medes Islands	W25 (2012)
Common Ravens (5)	ST-354 (1)	354	Barcelona province	W54 (2008)
	ST-952 (3)	8513	Barcelona province	W50 (2008), W51 (2008), W52 (2008)
	Singleton (1)	8514	Barcelona province	W53 (2008)
Fereal Pigeons (14)	ST-45 (7)	45	Barcelona	W32 (2015), W36 (2015), W37 (2015), W40 (2015), W43 (2015), W44 (2015)
		8512	Barcelona	W47 (2015)
	ST-179 (7)	179	Barcelona	W33 (2015)
		220	Barcelona	W41 (2015)
		2209	Barcelona	W34 (2015), W39 (2015), W46 (2015), W48 (2015), W49 (2015)
Northern shoveler (1)	Singleton (1)	996	Ebro Delta	W02 (2008)
White storks (2)	ST-354 (2)	354	Lleida	W55 (2011), W56 (2011)


*In brackets, number of strains.*

Isolates were recovered from stock cultures stored at -80°C in cryovials containing Brain Heart Infusion broth (BHI; Merck KGaA, Darmstadt, Germany) supplemented with 20% glycerol. Fresh cultures were obtained by streaking a loop of the frozen stock cultures onto blood agar plates (BioMérieux, Marcy l’Etoile, France); plates were incubated at 37°C for 48 h under a microaerobic atmosphere (85% N_2_, 10% CO_2_, 5% O_2_; Anaerocultaaa, Merck, Darmstadt, Germany).

### Species Identification and Antimicrobial Susceptibility Testing

Confirmation of *C. jejuni* strains was performed by conventional species-specific PCR using primers targeting the lipid A gene *lpxA* (Klena et al., 2004) and Matrix-Assisted Laser Desorption/Ionization-Time of flight (MALDI-TOF) Mass Spectrometry (Bruker Daltonics). Susceptibility to 12 antimicrobial agents was assessed by the disk diffusion method according to the Clinical Laboratory and Standard Institute [M100-S26; (CLSI, 2016)] using Mueller-Hinton medium supplemented with 5% defibrinated sheep blood (BioMérieux). The antimicrobials tested were: ampicillin (10 μg) (AMP), amoxicillin-clavulanic acid (30 μg) (AMC), imipenem (10 μg) (IMP), tetracycline (30 μg) (TET), erythromycin (15 μg) (ERY), ciprofloxacin (5 μg) (CIP), nalidixic acid (30 μg) (NAL), gentamicin (10 μg) (G), streptomycin (10 μg) (S), kanamycin (30 μg) (K), chloramphenicol (30 μg) (CHL), and fosfomycin (200 μg) (FOS). The breakpoints were performed following the CLSI criteria except for TET, ERY and CIP where the European Committee on Antimicrobial Susceptibility Testing (EUCAST) breakpoints criteria was used (Supplementary Table S1). All the clinical intermediate values were considered as resistant. The strains that showed resistance to three or more classes of antimicrobial agents were considered as multidrug-resistant (MDR) (Schwarz et al., 2010).

### Pulsed Field Gel Electrophoresis (PFGE) and Multi Locus Sequence Typing (MLST)

The PFGE was performed following the Standard Operating Procedure of PulseNet^1^ for *C. jejuni*. Genomic DNA was digested with SmaI and KpnI restriction enzymes (Sigma-Aldrich, United States). Electrophoresis was performed in a CHEF-DR III System (Bio-Rad Laboratories, Hercules, CA, United States). We performed a comparison analysis of PFGE profiles using the BioNumerics v7.6.3 software (AppliedMaths, Sint-Martens-Latem, Belgium). Similarity matrices were calculated by the Dice coefficient (2% optimization and 1% position tolerance) and dendrograms were constructed using the UPGMA method using the cited BioNumerics v7.6.3 software. Strains with a similarity ≥ 95% were considered as the same pulsotype, that means there are not more than two bands of difference.

*Campylobacter jejuni* strains were typed by MLST according to the procedures of PubMLST^2^. DNA was obtained using GenElute^TM^ Bacterial Genomic DNA Kit (Sigma-Aldrich, United States). Sanger sequence data were analyzed using BioNumerics v7.6.3 software. Alleles and sequence types (STs) were assigned based on the MLST scheme provided on the *Campylobacter* PubMLST^2^ database. Novel alleles and STs were submitted to the database.

To represent the relationship among *Campylobacter* strains, we generated a complete minimum spanning tree (MST) using the BioNumerics v7.6.3 software.

### Virulence-Associated Genes Detection

The 150 *C. jejuni* strains were tested by PCR for the presence of 8 genes encoding putative virulence factors. These included genes related to adhesion and colonization (*cadF*), invasion (*ciaB, virB11, htrA*, and *hcp*), and cytotoxin production (*cdtA, cdtB*, and *cdtC*). Genomic DNA was extracted by standard procedures using the InstaGene matrix Kit (Bio-Rad Laboratories). PCR reactions (PCR Master Mix x2, Thermo Scientific) were performed using 35 ng of DNA as a template and the specific primers indicated in Supplementary Table S2. As internal control of the PCR reaction, primers for the amplification of the housekeeping gene *gltA* were included in the PCR mixtures.

### Statistical Analysis

The virulence-associated genes data was analyzed using Pearson’s chi-squared test (R Studio software). *p* < 0.05 was considered statistically significant.

## Results and Discussion

### Genetic Diversity of *C. jejuni* Strains From the Three Different Ecological Niches

A collection of 150 *C. jejuni* isolates recovered from fecal samples from different ecological niches [50 isolates/each: human patients suffering gastroenteritis (H), broilers (B), and wild birds (W)] have been the focus of the study. The clonal relationship of the whole collection was determined by PFGE profiling. Four strains were non-typeable because of DNA smearing: one human (H49), one wild bird (W10), and two broiler (B24, B50) strains. As expected, a high clonal diversity was found among the 146 typeable strains. Genotyping using the SmaI restriction enzyme resulting 120 pulsotypes and 12 clones. To increase the clonal discrimination, the secondary KpnI enzyme was used (On et al., 1998). The combined analysis of SmaI and KpnI-PFGE banding patterns resulted in a wider clonal diversity with 137 pulsotypes and 4 clones (Supplementary Figure S1). The highest clonal diversity was found in the human population where no strains with the same pulsotype were found, consistent with the fact that human samples were not chosen in the context of an outbreak. By contrast, 13 strains, 6 from broilers and 7 from wild birds, were grouped in four clones (similarity ≥ 95%). Clone 1 included 6 broiler strains (B52–B57) from 2 different farms (5 from EU and 1 from CT) belonging to the same broiler company (**Table 1**). The five strains (B52–B56) from farm EU were recovered from broilers included in the same flock. This possible flock colonization is not a rare case and has also previously been reported by other authors (Ridley et al., 2011). Also, the recovery of the same clone in broilers from two different farms may be due to cross contamination between farms belonging to the same broiler company. Clone 2, included three strains (W06–W08) from Audouin’s gulls (*Larus audouinii*) sampled at Alboran Island at the same breeding season, and Clones 3 and 4, both with two strains, W43 and W44 from pigeons (*Columba livia*, Barcelona) and W50 and W52 from common ravens (*Corvus corax*) (Sabadell, Barcelona province), respectively. It is not surprising the finding of different strains from the same host species belonging to the same clone, since samples were collected from different birds belonging to the same colony during the same time period.

The remaining PFGE patterns from the three different niches were scattered along the dendrogram, although different clusters could be observed (Supplementary Figure S1). Overall, human and broiler strains frequently grouped together at different similarity levels. On the contrary, a marked host specificity was found within some specific genus of wild birds (*Columba, Corvus*, and *Ciconia*) as previously described (Griekspoor et al., 2013). Thus, pigeons’ strains were grouped in different clusters with a similarity ranging from 65 to 80%; the three ravens’ strains (two of them constitute the Clone 4) clustered with a similarity of 82% and the two storks clustered with a broiler strain with a 65% similarity. Finally, the northern shoveler strain showed low similarity (≤ 50%) with all other wild birds strains (Supplementary Figure S1). Gulls strains were scattered along the dendrogram but some clusters were also defined. Interestingly, a cluster containing strains from two different gull species had a similarity of 65%. These gulls were from different geographical locations (Medes Islands, Ebro Delta, and Alboran Island). This fact has already been described by Griekspoor et al. (2013) who found a high similarity between strains from the same or closely related bird species from different geographical areas (Sweden, Australia, and United Kingdom). Notably, the gull isolate W30 cluster together with several pigeon isolates (W32, W36, W37). Since all those isolates were recovered from birds at the same geographical area (Barcelona), these data suggest that both the gull and the pigeons have a common source of infection or it is a consequence of gulls being pigeons predators.

### MLST Analysis and Population Structure

The MLST analysis corroborates the genetic diversity observed by PFGE typing among the *C. jejuni* strains and confirms a closer relationship between human and broiler strains.

Among the 150 strains studied, 64 different STs grouped in 21 clonal complexes (ST-complexes) and 12 singletons (S) were recognized (**Table 3** and Supplementary Figure S2). Six novel STs were identified: ST-8479 (human), ST-8498 (broiler), ST-8511, ST-8512, ST-8513, and ST-8514 (wild birds). In this study, the ST-21 (24 strains), ST-1275 (16 strains), and ST-45 (13 strains) clonal complexes were the most frequent. Strains from the ST-1275 complex were only isolated from two different gull species. The ST-21 complex was found in humans (16 strains) and broilers (8 strains), whereas the ST-45 was found in the three environments studied. Thus, the ST-45 complex was predominant in wild birds (8 strains), followed by broilers (4 strains) and by humans (1 strain). Both, the ST-21 and ST-45 complexes are described as multihost genotypes and have been isolated from a wide variety of agricultural and environmental sources (Sopwith et al., 2006; French et al., 2009; Sheppard et al., 2011; Colles and Maiden, 2012).

**Table 3:** Clonal complex (ST-complex), sequence type (ST), virulence factors profile (VF), and antimicrobial resistance profile (AMR) found in 150 *C. jejuni* strains from humans, broilers, and wild birds niches.

			Humans		Broilers		Wild Birds	
ST complexes	ST		VF^a^	AMR^b^	VF	AMR	VF	AMR
ST-21 complex (24)	19	(1)	#2	M (1)				
	21	(8)	#2	M (1) I (2) J (1) T (1)	#2	M (2) O (1)		
	50	(8)	#2	M(4) K (1)	#2	M (1) K (2)		
	883	(4)	#2	M (1) J (1)	#1	I (1)		
					#2	I (1)		
	1214	(1)	#2	J (1)				
	3769	(1)	#2	M (1)				
	4664	(1)	#2	B (1)				
ST-42 complex (2)	459	(1)	#2	M (1)				
	4016	(1)	#2	E (1)				
ST-45 complex (13)	45	(10)	#2	K (1)	#2	M (1) G (1)	#2	A (3) C (3) I (1)
	137	(1)			#2	P (1)		
	652	(1)			#2	P (1)		
	8512	(1)					#2	A (1)
ST-48 complex (3)	48	(3)	#5	M (1)	#9	M (1)	#14	M (1)
ST-49 complex (1)	49	(1)	#2	M (1)				
ST-52 complex (1)	52	(1)	#2	H (1)				
ST-61 complex (5)	61	(5)	#6	M (2) A (1) E (1)				
			#12	B (1)				
ST-179 complex (7)	179	(1)					#5	A (1)
	220	(1)					#3	D (1)
	2209	(5)					#2	A (1)
							#5	A (2) C (2)
ST-206 complex (6)	46	(1)			#2	M (1)		
	227	(1)	#2	A (1)				
	572	(4)	#5	M (4)				
ST-257 complex (12)	257	(3)	#2	M (1) N (1) P(1)				
	367	(6)			#2	M (6)		
	2254	(3)	#2	M (2)	#2	I (1)		
ST-283 complex (1)	267	(1)			#2	M (1)		
ST-353 complex (9)	5	(2)			#1	I (1)		
					#3^∗^	I (1)		
	353	(1)	#1^∗^	K (1)				
	356	(1)			#2	M (1)		
	400	(5)	#3	M (1)	#3	I (3) M (1)		
ST-354 complex (9)	354	(8)	#2	M (2)	#2	M (2)	#2	M (2) I (2)
	8498	(1)			#2	M (1)		
ST-443 complex (2)	51	(1)	#2	M (1)				
	5799	(1)	#2	M (1)				
ST-446 complex (1)	3552	(1)					#1	M (1)
ST-464 complex (3)	464	(3)	#1	F (1)	#2	M (1)		
					#1	M (1)		
ST-574 complex (2)	305	(2)			#2	M (1) I (1)		
ST-607 complex (8)	607	(2)			#1	M (1) I (1)		
	904	(3)	#1	M (1)	#1	M (2)		
	1707	(1)			#1	M (1)		
	7110	(2)			#2	M (1)		
					#1	I (1)		
ST-952 complex (3)	8513	(3)					#10	A (2) C (1)
ST-1034 complex (1)	4001	(1)					#13	L (1)
ST-1275 complex (16)	637	(2)					#4	A (1) B (1)
	1223	(5)					#4	C (1)
							#7	A (2) C (1)
							#15	C (1)
	1268	(1)					#4	B (1)
	1275	(3)					#4	B (2)
							#8	B (1)
	1292	(2)					#4	C (1)
							#8	C (1)
	3049	(1)					#4	A (1)
	3629	(1)					#4	E (1)
	8511	(1)					#4	B (1)
Singletons	441	(3)	#2	R (1)	#5	P (1)		
			#11	J (1)				
	531	(2)	#3	M (1)	#1	M (1)		
	996	(1)					#16	A (1)
	1261	(1)					#2	A (1)
	1343	(2)					#2	A (2)
	1710	(4)	#1	M (1)	#1	M (1) Q (1)		
					#3	P (1)		
	2331	(1)			#1	M (1)		
	2351	(1)					#2	C (1)
	4355	(3)					#2	A (3)
	7114	(1)			#2	T (1)		
	8479	(1)	#1	M (1)				
	8514	(1)					#7	S (1)


*^*a*^The VF profile is described in **Figure 3**. ^*b*^The AMR profile is described in **Figure 2**. ^∗^virB11 positive. In brackets, number of strains.*

**FIGURE 1:**
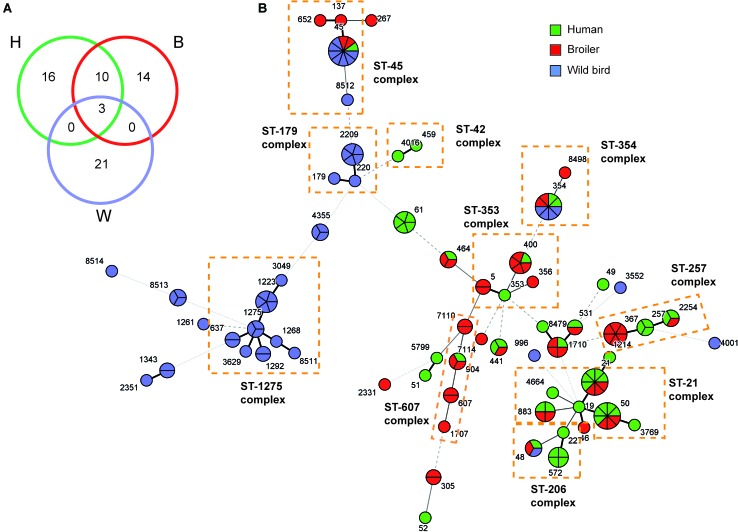
Minimum spanning tree showing the distribution of the *C. jejuni* strains. **(A)** The number of STs shared among the three niches was represented by a Venn-diagram. **(B)** Minimum spanning tree (MST) of *C. jejuni* sequence types (STs) based on allele numbers. Each circle corresponds to a ST, being its size proportional to the number of strains within it. The ST of each strain are indicated in different color attending to the origin of the strain: green for humans (H), red for broilers (B), and blue for wild birds (W). The thickness and the dotting of the lines indicate the distance between STs. A thicker line denotes closer distance than a thin line and a thin line denotes closer distance than a dotted line. The ST-complexes including several STs are indicated in dotted color lines. The MST analysis was carried out by BioNumerics v7.6.

The relationship among the three studied populations is reflected in a minimum spanning tree diagram (**Figure 1**). Three STs (ST-45, ST-48, and ST-354) were found in all three niches representing 14% (21/150) of the 150 studied strains. The spanning tree diagram distributes the strains in two main clusters, characterized – with some exceptions – by its ecology. The first cluster, in the left side of the **Figure 1B**, grouped STs from wild birds, whereas the second cluster (right side) agglutinate most human and broiler STs. This distribution clearly establishes a closer relationship between broiler and human strains, consistent with broiler meat being the most frequent source of *Campylobacter* human infection (EFSA-ECDC, 2016; Ramonaite et al., 2017). This can also be observed when looking at the number of STs shared among the different niches (**Figure 1**): 10 STs are shared between human and broiler strains, only 3 in the 3 niches, and no ST is shared among wild birds and either humans or broilers.

From the first cluster (STs from wild birds), the ST-179 and ST-1275 complexes were mainly found in pigeons and seagulls, respectively. These data support that *Campylobacter* genotypes isolated from non-agricultural sources, such as wild birds, are found only rarely among broilers (Griekspoor et al., 2010). However, these clonal complexes have also been recovered from stool samples from humans with sporadic cases of gastroenteritis (Hughes et al., 2009). STs from the ST-179 complex have been isolated from environmental sources, specifically from the sand of bathing beaches in the United Kingdom, presumably ultimately coming from wild birds (Dingle et al., 2001). The ST-1275 complex has also been found in several wild bird species such as pigeons (Sheppard et al., 2009). Certain host specificity was found among the ST-complexes present in different wild birds. Hence, the ST-1275 complex was predominant in gulls, the ST-179 complex in pigeons and the ST-952 complex in ravens (**Table 2**).

With regards to the second cluster (STs from human and broiler strains), five ST- complexes were found as predominant (ST-21, ST-206, ST-257, ST-353, and ST-607). These clonal complexes have been previously associated with human infections, poultry, other farm animals, and environmental samples (Colles and Maiden, 2012; Cody et al., 2015; Ramonaite et al., 2017). Overall, a great diversity of ST-complexes was found among both human and broiler strains. A total of 16 STs were found in humans and 14 in broilers (**Figure 1A**). Strains from Clones 1, 2, 3, and 4 by PFGE belong to ST-367, ST-4355, ST-45, and ST-8513, respectively, while the remaining strains showed unrelated PFGE pattern.

### Antimicrobial Susceptibility

**FIGURE 2:**
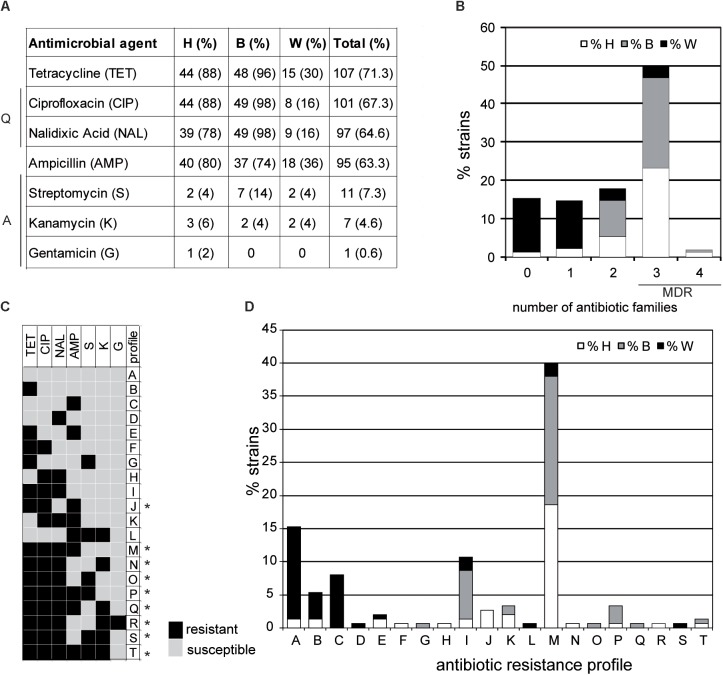
Antimicrobial resistance in *C. jejuni* studied strains. **(A)** Absolute and relative values of antimicrobial resistance in human (H), broiler (B), and wild bird (W) strains. Antibiotic families (Q: quinolones and A: aminoglycosides) are indicated. All strains were susceptible to amoxicillin-clavulanic acid, imipenem, erythromycin, chloramphenicol, and fosfomycin. **(B)** Percentage of resistant strains to 0 to 4 families of antibiotics. MDR indicates multidrug-resistant strains. **(C)** Antimicrobial resistance profiles obtained. Asterisks indicate the MDR profiles. **(D)** Percentage of the different antimicrobial resistance profiles in each subgroup, as defined in **(C)**.

Antimicrobial susceptibility was tested for all the isolates and antimicrobial resistance (AMR) profiles were defined (**Figure 2**). In agreement with the increase in the occurrence of AMR and MDR detected in *Campylobacter* strains in many countries (Luangtongkum et al., 2009), a high frequency of AMR was detected among the strains of our collection. A total of 127 strains (84.6%) were resistant to one or more antimicrobial agents. The highest percentage of antimicrobial resistance was found for tetracycline (71.3%), quinolones [ciprofloxacin (67.3%), nalidixic acid (64.6%)], and ampicillin (63.3%) (**Figure 2A**). In agreement with the genotyping data, the antimicrobial susceptibility describes important differences between wild bird strains and the clustered broiler and human strains, with the occurrence of antibiotic resistance much lower among wild bird strains. In our strain collection, 23 strains were susceptible to all antimicrobial agents tested. From those, 21 strains were recovered from wild birds and the other two from human samples. Moreover, the occurrence of tetracycline, quinolones, and ampicillin resistance was higher in human and broiler strains compared to wild birds. In contrast, the low occurrence resistance to aminoglycosides is shared among strains from the three niches. In our study, 7.3 and 4.6% of the strains were resistant to streptomycin and kanamycin, respectively, whereas only one strain from a human sample was resistant to gentamicin.

The high occurrence of resistance to some antibiotics cannot be accounted to the non-therapeutic use of antimicrobial drugs in poultry production since this practice was banned in the EU in 2006. The therapeutic antibiotics used in poultry are mainly quinolones (enrofloxacin), tetracyclines (doxycycline), penicillins (amoxicillin), and macrolides (erythromycin, tylosin), which is reflected – in general – with the occurrence of antibiotic resistance detected. Also in agreement with the detected occurrence, the aminoglycosides tested (streptomycin, kanamycin, and gentamicin) are not of common use for treatment of enteric diseases in poultry (Cantero et al., 2018). All strains were susceptible to amoxicillin-clavulanic acid, imipenem, erythromycin, chloramphenicol, and fosfomycin. The rare occurrence of imipenem, erythromycin, and amoxicillin-clavulanic acid resistance among *Campylobacter* strains has been previously described (Gallay et al., 2007; Deckert et al., 2010; Deng et al., 2015). In contrast, resistance to fosfomycin has been detected in *C. jejuni* strains (Novoa-Farías et al., 2016) as well as to chloramphenicol in *C. coli* strains isolated from humans and broilers (Yang et al., 2017).

The MDR, defined as resistance to three or more families of antimicrobial agents (Schwarz et al., 2010), has increased worldwide among *C*. *jejuni* strains (Giacomelli et al., 2014). In this study, 49.3% of the strains were MDR (**Figure 2B**). The most frequent MDR profile (M profile, TET-NAL-CIP-AMP) was detected in 40% of the strains, being mostly identified in humans (23.3%) and broilers (23.3%) (**Figures 2C,D**). Only five strains from wild birds were MDR, four of them with the above mentioned M profile and one with the S profile (TET-NAL-CIP-S-K). Those MDR strains were recovered from birds with insalubrious feeding habits (feeding on refuse dumps and carrion) such as gulls, storks, and ravens (Ramos et al., 2010).

These MDR strains were included in five out of the six predominant ST-complexes found in humans and broilers (ST-21, ST-206, ST-257, ST-354, and ST-607) (**Table 3**). Interestingly, the percentage of MDR among strains of ST-353 complex, also defined as predominant in humans and broilers, was much lower (33.3 %). No MDR strains have been found among the two ST- complexes exclusive for wild birds (ST-179 and ST-1275). It should be highlighted that there is a high variability in the AMR profile among genetically closely related strains. For instance, within the ST-45 and ST-61, strains susceptible to all antimicrobial agents tested and MDR were found. These suggest a rapid acquisition of antibiotic resistance determinants among *C. jejuni* strains. Accordingly, transfer of antibiotic resistance genes by mobile genetic elements, such as plasmids and transposons that can help acquisition and diffusion of drug resistance, has been described (Bennett, 2008).

### Prevalence and Distribution of Virulence-Associated Genes Among the *C. jejuni* Strains

**FIGURE 3:**
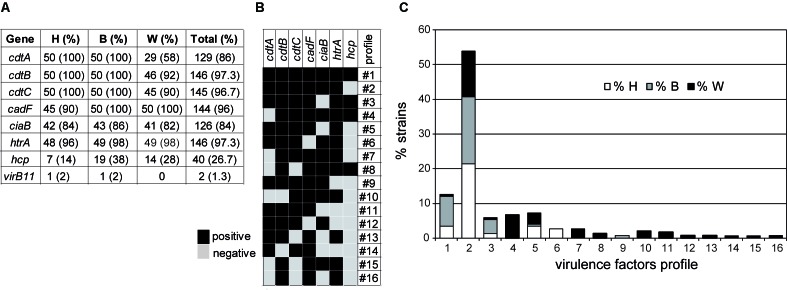
Virulence factors in *C. jejuni* studied strains. **(A)** Absolute and relative values of the virulence factors found in human (H), broiler (B), and wild bird (W) strains. **(B)** Virulence factors profiles obtained. **(C)** Percentages of the different virulence factors profiles in each subgroup, as defined in **(B)**.

The presence of genes coding for putative virulence factors in the 150 *C. jejuni* strains have been tested by PCR. The genes detected were: (1) *cdtA, cdtB*, and *cdtC* genes, conforming a polycistronic operon, coding for synthesis and deliver of the cytolethal distending toxin that causes host cell cycle arrest; (2) three genes involved in adherence and invasion of host cells: *cadF* coding for a putative OmpA-like protein that mediates bacterial adhesion by binding to host fibronectin, *ciaB* coding for an invasive antigen that translocate into the cytoplasm of host cells facilitating the *C. jejuni* invasion, and *htrA* coding for a serine-protease that contributes to stress tolerance and with ability to cleave E-cadherin; (3) the *hcp* gene coding for a host surface adhesion protein that is a component of a type 6 secretion system (T6SS) that has been associated with virulence; and (4) the *virB11*, a gene located in the virulence related plasmid pVir (Bacon et al., 2002; Tracz et al., 2005; Cróinín and Backert, 2012; Bleumink-Pluym et al., 2013). The results showed a high prevalence of six out of eight genes tested (**Figure 3**). Remarkably, significant differences exist in the prevalence of some of the genes when comparing among the strains from different origin. A different distribution of the *cdtA, cdtB*, and *cdtC* genes was detected among the three subgroups of strains. The three genes were present in all the human and broiler strains whereas 46% of the wild bird strains were negative for at least one of the *cdt* genes (Supplementary Figure S2). The fact that many strains (23) lacked one or two of the three open reading frames (ORF) present in the polycistronic *cdtABC* operon is an interesting phenomenon that has been reported earlier (Bang et al., 2003; Koolman et al., 2015). Remarkably, the *cdtA* gene had the more uneven distribution since it was detected in all human and broilers strains, while it was only present in 58% of the wild bird strains. Among the 23 strains lacking at least one *cdt* gene, 14 lacked *cdtA*, 1 *cdtB*, 1 *cdtC*, 3 *cdtAB*, and 4 *cdtAC*. The negative PCR-amplification of one or two of the three ORF present in the *cdtABC* operon, when using primers designed from the 81-176 genome sequence (Supplementary Table S2), may indicate significant differences among alleles from different strains. Our results suggest that: (1) a high diversity of *cdt* alleles exists among *C. jejuni* strains found in natural environments (wild bird) and (2) the *cdt* alleles promoting efficient broiler colonization are much less diverse. *cdtA* and *cdtC* are involved in the binding to the target host cells allowing internalization of the *cdtB* toxin. Therefore, we hypothesize that different *cdtA* might have distinct target molecules during tissue recognition and consequently might play a relevant role defining the host susceptibility of *C. jejuni* strains. Interesting observations can be made when having a closer look to the phylogenetic and ecological relationships of the strains that were negative for any of the *cdt* genes (**Table 3** and Supplementary Figure S2): (1) all 16 strains belonging to the ST-1275 complex and different PFGE pattern were *cdtA* negative and were isolated from the two different seagull species located in three geographically distinct colonies along the western Mediterranean coast; three of them were also *cdtC*-negative. From these seagulls’ colonies, *cdtABC*^+^ strains, belonging to diverse ST-complexes distinct to ST-1275, were also isolated; (2) all three strains belonging to ST-952 complex are *cdtAB* negative but two share the same PFGE pattern (W50 and W52), these strains were isolated from commons ravens. The other two ravens’ strains are *cdtA* negative (W53, singleton ST-8514) and *cdtABC*^+^ (W54, ST-354 complex), respectively; (3) the ST-48 complex includes three strains, belonging each one to a different niche, the broiler (B09) and human (H38) strains are *cdtABC*^+^ and showed different PFGE pattern, whereas the wild bird strain (W29) from a Yellow-legged gull is *cdtB* negative; (4) from the wild bird subgroup, all the strains from pigeons and storks, birds that are more in contact with the human population, are positive for all three *cdt* genes; and (5) the unique *cdtC*^-^ strain belongs to the ST-1034 complex. The relevant diversity among *cdtABC* found among wild bird strains and the apparently highly conserved distribution among human and broiler strains might indicate that the specific sequence of certain *cdt* alleles can be related to the ability to colonize different hosts. Moreover, the fact that there are differences in the presence of *cdt* alleles among strains from the same ST-complex and/or ST indicates the high plasticity of this genetic locus (the *cdtABC* operon).

Regarding the genes coding for putative factors involved in host cell invasion, only *cadF* showed an uneven prevalence distribution among strains from the different niches. The tested *cadF* allele was present in all broiler and wild bird strains and in 45 out of 50 human strains. This result suggests that either some strains lack the *cadF* gene or carry a *cadF* allele with significant differences from the *cadF*_81-176_ allele chosen as template in this study (Supplementary Table S2). Interestingly, all the *cadF* negative strains belong to ST-61, which is often found in cattle/bovine reservoirs (Kwan et al., 2008). Our results suggest that the presence of the *cadF* tested may not be essential for the ability of *Campylobacter* to cause infection in humans. It cannot be disregarded that the five *cadF^-^* strains carry a different *cadF* allele not detected with the primers used (Supplementary Table S2). In a recent report studying the distribution of virulence-associated genes in a collection of 24 *Campylobacter* strains, *cadF* was detected in all *C. jejuni* strains whereas some *C. coli* from both humans and broilers were negative for this gene (Koolman et al., 2015).

No significant differences exist when comparing the prevalence of the *ciaB* gene among the three populations. The *ciaB* negative strains are the majority for certain ST-complexes such as ST-48, ST-179, ST-353, ST-206, and two singletons (ST-441, ST-1710). However, we also found *ciaB* negative strains belonging to ST-complex where most strains are positive for this genetic locus. Only four strains were negative for *htrA*, two from humans (ST-61 and ST-441), one from a broiler (ST-48), and one from a wild bird (ST-48).

The presence of the *hcp* gene is a hallmark of the ability of *C. jejuni* to express a T6SS (Lindmark et al., 2009). A functional T6SS has been identified in some *C. jejuni* strains, those carrying the integrative element CJIE3. Its presence had been related to the ability to infect humans and cause disease by influencing cell adhesion and cytotoxicity toward erythrocytes (Lertpiriyapong et al., 2012; Bleumink-Pluym et al., 2013; Harrison et al., 2014). Notably, the *hcp* gene was detected in a low proportion in all three populations. The detection frequency was in a range similar to that described in previous reports (Harrison et al., 2014; Siddiqui et al., 2015). Surprisingly, our data indicate that *hcp* is significantly less represented among the human strains (14%) as compared to broiler (38%) and wild bird (28%) strains (**Figure 3A**). Interestingly, the *hcp*-positive clones are mostly distributed among certain ST-complexes: ST-1275 (12 out of 16 are positive), ST-607 (7/8), ST-353 (8/9), ST-464 (2/3), and ST-446 (1/1). The differential prevalence of *hcp*, with lower prevalence among human strains, suggests that T6SS does not promote human infection. The last putative virulence factor encoding gene tested was *virB11*. Previous reports have argued on the contribution of the pVir plasmid in the virulence potential of *C. jejuni* (Tracz et al., 2005; Louwen et al., 2006). Among our strains the prevalence of the *virB11* gene is very low since only two strains, one from human and one from broiler, were positive for the tested *virB11* allele, suggesting that the pVir plasmid is not required for *C. jejuni* to either colonize birds or infect humans.

Virulence profiles were defined attending to the presence/absence of virulence-related genes of chromosomal location (all genes except *virB11*) (**Figure 3**). The virulence profile #2 (*cadF*^+^, *cdt*^+^, *htrA*^+^, *ciaB*^+^) is overrepresented among the collection, being detected in a 54% of the strains and being the most prevalent in all three subgroups (human, broiler and wild birds). In addition to profile #2, only three more virulence profiles (#1, #3, #5) were detected in all the three subgroups. The highest variability was found among the wild bird strains since up to 14 different profiles were identified, in contrast to human and broiler strains where five profiles were found within each subgroup. From the 14 profiles described in wild birds, 10 were only found in this subgroup. One virulence profile (#6, *cdt*^+^, *cadF*^-^, *ciaB*^+^, *htrA*^+^ *hcp*^-^) was only found among human strains, being detected in four clinical strains belonging to ST-61, but with different PFGE patterns. Moreover the profile #9 has only been detected among broiler strains.

## Conclusion

In this report, a comparative study has been performed among *C. jejuni* strains from three different sources: humans, broilers, and wild birds from Catalonia. As expected, by PFGE a great genetic diversity was observed among all included strains, with the exception of a few clones. In our *Campylobacter* population, the ST-45, ST-48, and ST-354 were present in all three niches; the ST-21 complex was the predominant in human and the ST-1275 complex in wild birds, while in broilers different complexes: ST-21, ST-257, ST-353, and ST-607 were present equally. MLST analysis clearly distinguished the wild bird *Campylobacter* population from that of humans and chickens, suggesting that certain host specificity may exist among *C. jejuni* clonal complexes. The drug antimicrobial profiles show an overall high percentage of MDR strains (49%). Again, a closer relationship between human and broiler strains was detected. A similar high percentage of MDR strains (72%) was detected among human and broiler strains whereas the percentage among wild bird strains was much lower (8%). This discrepancy in MDR prevalence correlates with the differential antibiotic pressure. The broiler and human strains are under a high antibiotic pressure due to the use of antimicrobial drugs whereas the selective pressure is barely inexistent in wild bird population. The presence of several putative virulence genes have been detected by PCR. The genes *cadF, ciaB, htrA*, and the operon *cdtABC*, coding for proteins presumably involved in bacterial adherence, invasion of epithelial cells and toxin production, are highly prevalent among *Campylobacter* strains. Correlation between certain virulence factors profiles and specific STs has been detected, which may have ecological implications. For instance, our data indicates a great diversity in *cdtA* alleles among wild bird strains as compared with broiler and human strains, suggesting that colonization of specific hosts might be promoted by certain *cdtA* variants.

All the genotype- and phenotype-based analysis indicate that most strains isolated from wild birds form a cluster clearly differentiated from those strains isolated from broilers and humans. Nonetheless, some wild bird strains belong to clonal complexes also detected among broiler or human strains suggesting a reverse zoonosis transmission, most likely consequence of the scavenging feeding habitats of the studied birds. Overall, our report provides new insights into the distribution of circulating *C. jejuni* strains among different ecological niches.

## Author Contributions

YI-T performed the investigation, formal analysis, and statistical analysis, and wrote the manuscript. PG performed the investigation, formal analysis, and statistical analysis. TL and CMu performed the conception and design of the study, and revised the manuscript. MC-C and FN performed the formal analysis, statistical analysis, conception, and design of the study, and revised the manuscript. EM, CMa, and CB performed the formal analysis, statistical analysis, conception, and design of the study, and wrote the manuscript.

## Conflict of Interest Statement

The authors declare that the research was conducted in the absence of any commercial or financial relationships that could be construed as a potential conflict of interest.

## Footnotes

1 http://www.pulsenetinternational.org/protocols/pfge

2 http://pubmlst.org/campylobacter
