# Supplementary material for: Population Structure, Antimicrobial Resistance, and Virulence-Associated Genes in Campylobacter jejuni Isolated From Three Ecological Niches: Gastroenteritis Patients, Broilers, and Wild Birds
Source: Front Microbiol. 2018 Aug 2;9:1676. doi: 10.3389/fmicb.2018.01676 (PMC6083060; doi:10.3389/fmicb.2018.01676)
Supplement: Supplementary file 1 [file Data_Sheet_1.PDF]

Supporting information (Iglesias-Torrens et al.)

Figure S1. PFGE combined dendogram of SmaI and KpnI patterns of *C. jejuni* strains. Color of the branches indicates different host niches (green for humans, red for broilers and blue for wild birds). The clones are framed and named. Black and blue vertical lines indicate the percentage of homology (95% and 65%) cited in the text.

Figure S2. Virulence factors profiles, STs and ST-complexes of the 150 *C. jejuni* strains, ordered for their ST-complex. The species of the wild bird strains are indicated (YLGB: yellow-legged gulls from Barcelona; YLGM: yellow-legged gulls from Medes Islands; AGD: Audouin's gulls from Ebro Delta: AGA: Audouin's gulls from Alboran Islands; FP: feral pigeons; CR: common ravens; WS: white storks; NS: northern shoveler: *Spatula clypeata*).

Table S1. Breakpoints used in the antimicrobial susceptibility tests.

Table S2. Primers used in this work

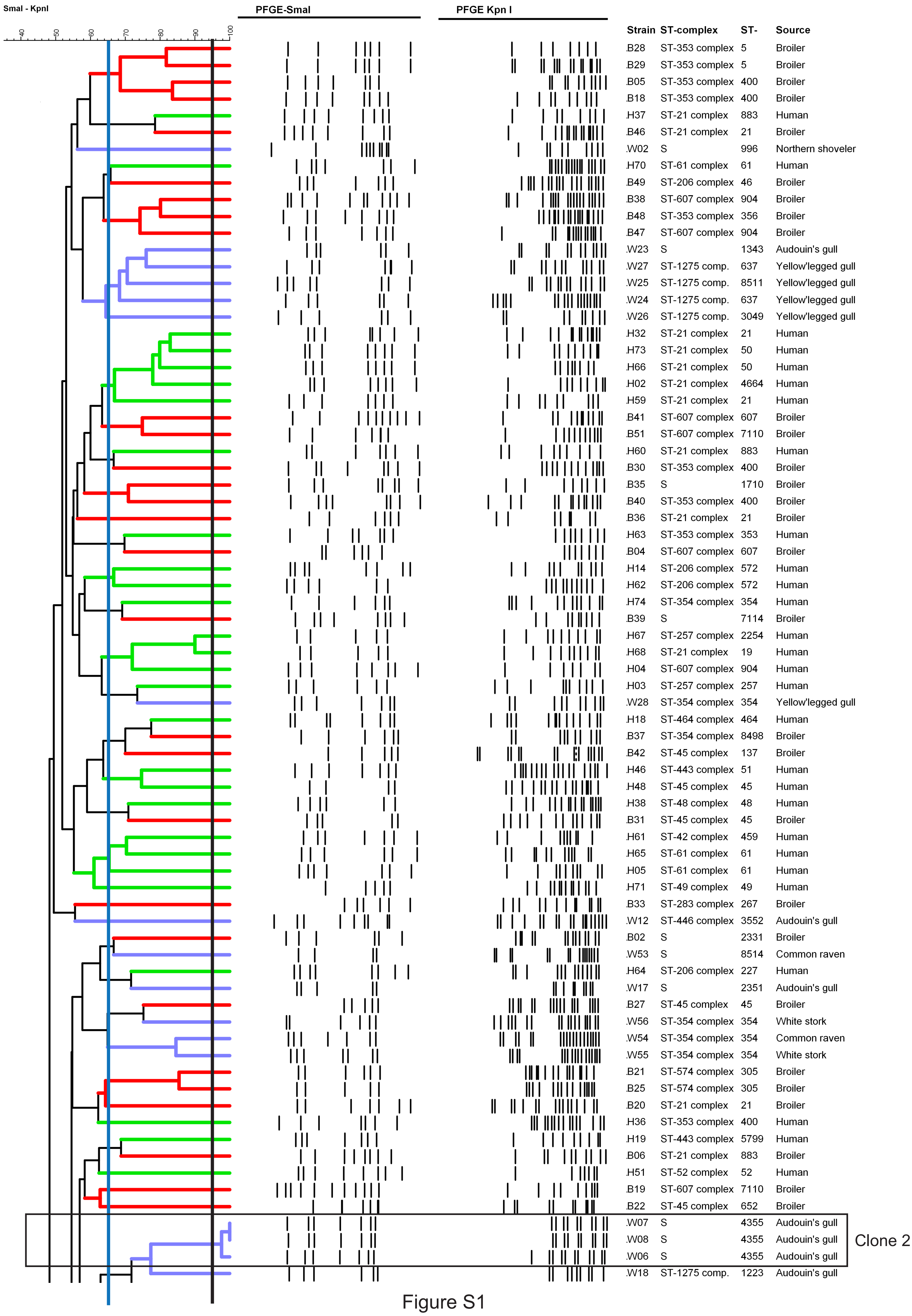

Strain	ST-complex	ST-	Source	
B28	ST-353 complex	5	Broiler	
B29	ST-353 complex	5	Broiler	
B05	ST-353 complex	400	Broiler	
B18	ST-353 complex	400	Broiler	
H37	ST-21 complex	883	Human	
B46	ST-21 complex	21	Broiler	
W02	S	996	Northern shoveler	
H70	ST-61 complex	61	Human	
B49	ST-206 complex	46	Broiler	
B38	ST-607 complex	904	Broiler	
B48	ST-353 complex	356	Broiler	
B47	ST-607 complex	904	Broiler	
W23	S	1343	Audouin's gull	
W27	ST-1275 comp.	637	Yellow'legged gull	
W25	ST-1275 comp.	8511	Yellow'legged gull	
W24	ST-1275 comp.	637	Yellow'legged gull	
W26	ST-1275 comp.	3049	Yellow'legged gull	
H32	ST-21 complex	21	Human	
H73	ST-21 complex	50	Human	
H66	ST-21 complex	50	Human	
H02	ST-21 complex	4664	Human	
H59	ST-21 complex	21	Human	
B41	ST-607 complex	607	Broiler	
B51	ST-607 complex	7110	Broiler	
H60	ST-21 complex	883	Human	
B30	ST-353 complex	400	Broiler	
B35	S	1710	Broiler	
B40	ST-353 complex	400	Broiler	
B36	ST-21 complex	21	Broiler	
H63	ST-353 complex	353	Human	
B04	ST-607 complex	607	Broiler	
H14	ST-206 complex	572	Human	
H62	ST-206 complex	572	Human	
H74	ST-354 complex	354	Human	
B39	S	7114	Broiler	
H67	ST-257 complex	2254	Human	
H68	ST-21 complex	19	Human	
H04	ST-607 complex	904	Human	
H03	ST-257 complex	257	Human	
W28	ST-354 complex	354	Yellow'legged gull	
H18	ST-464 complex	464	Human	
B37	ST-354 complex	8498	Broiler	
B42	ST-45 complex	137	Broiler	
H46	ST-443 complex	51	Human	
H48	ST-45 complex	45	Human	
H38	ST-48 complex	48	Human	
B31	ST-45 complex	45	Broiler	
H61	ST-42 complex	459	Human	
H65	ST-61 complex	61	Human	
H05	ST-61 complex	61	Human	
H71	ST-49 complex	49	Human	
B33	ST-283 complex	267	Broiler	
W12	ST-446 complex	3552	Audouin's gull	
B02	S	2331	Broiler	
W53	S	8514	Common raven	
H64	ST-206 complex	227	Human	
W17	S	2351	Audouin's gull	
B27	ST-45 complex	45	Broiler	
W56	ST-354 complex	354	White stork	
W54	ST-354 complex	354	Common raven	
W55	ST-354 complex	354	White stork	
B21	ST-574 complex	305	Broiler	
B25	ST-574 complex	305	Broiler	
B20	ST-21 complex	21	Broiler	
H36	ST-353 complex	400	Human	
H19	ST-443 complex	5799	Human	
B06	ST-21 complex	883	Broiler	
H51	ST-52 complex	52	Human	
B19	ST-607 complex	7110	Broiler	
B22	ST-45 complex	652	Broiler	
W07	S	4355	Audouin's gull	Clone 2
W08	S	4355	Audouin's gull	Clone 2
W06	S	4355	Audouin's gull	Clone 2
W18	ST-1275 comp.	1223	Audouin's gull	

Figure S1

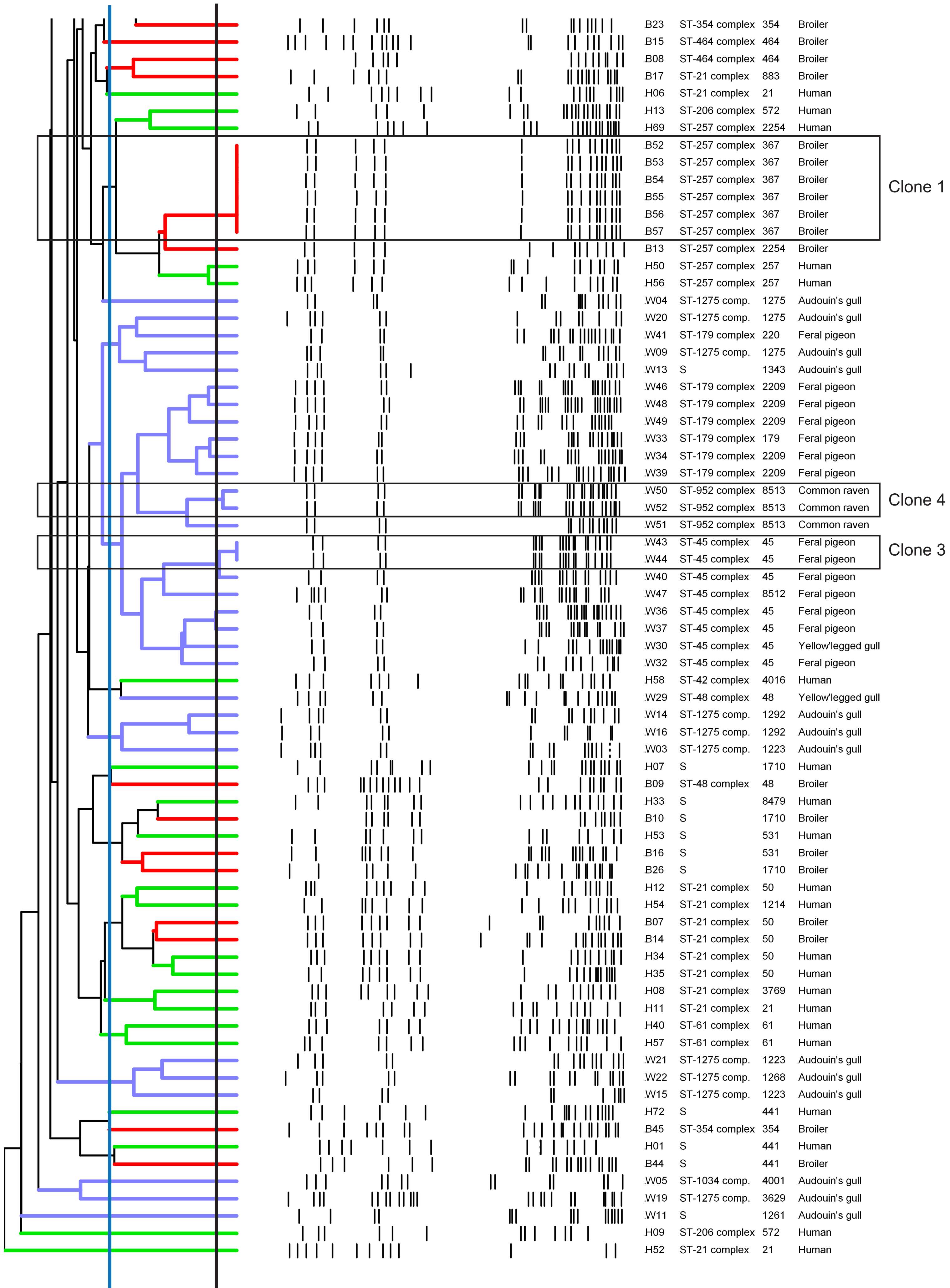
B23 ST-354 complex 354 Broiler
B15 ST-464 complex 464 Broiler
B08 ST-464 complex 464 Broiler
B17 ST-21 complex 883 Broiler
H06 ST-21 complex 21 Human
H13 ST-206 complex 572 Human
H69 ST-257 complex 2254 Human
B52 ST-257 complex 367 Broiler
B53 ST-257 complex 367 Broiler
B54 ST-257 complex 367 Broiler
B55 ST-257 complex 367 Broiler
B56 ST-257 complex 367 Broiler
B57 ST-257 complex 367 Broiler
Clone 1
B13 ST-257 complex 2254 Broiler
H50 ST-257 complex 257 Human
H56 ST-257 complex 257 Human
W04 ST-1275 comp. 1275 Audouin's gull
W20 ST-1275 comp. 1275 Audouin's gull
W41 ST-179 complex 220 Feral pigeon
W09 ST-1275 comp. 1275 Audouin's gull
W13 S 1343 Audouin's gull
W46 ST-179 complex 2209 Feral pigeon
W48 ST-179 complex 2209 Feral pigeon
W49 ST-179 complex 2209 Feral pigeon
W33 ST-179 complex 179 Feral pigeon
W34 ST-179 complex 2209 Feral pigeon
W39 ST-179 complex 2209 Feral pigeon
W50 ST-952 complex 8513 Common raven
W52 ST-952 complex 8513 Common raven
Clone 4
W51 ST-952 complex 8513 Common raven
W43 ST-45 complex 45 Feral pigeon
W44 ST-45 complex 45 Feral pigeon
Clone 3
W40 ST-45 complex 45 Feral pigeon
W47 ST-45 complex 8512 Feral pigeon
W36 ST-45 complex 45 Feral pigeon
W37 ST-45 complex 45 Feral pigeon
W30 ST-45 complex 45 Yellow'legged gull
W32 ST-45 complex 45 Feral pigeon
H58 ST-42 complex 4016 Human
W29 ST-48 complex 48 Yellow'legged gull
W14 ST-1275 comp. 1292 Audouin's gull
W16 ST-1275 comp. 1292 Audouin's gull
W03 ST-1275 comp. 1223 Audouin's gull
H07 S 1710 Human
B09 ST-48 complex 48 Broiler
H33 S 8479 Human
B10 S 1710 Broiler
H53 S 531 Human
B16 S 531 Broiler
B26 S 1710 Broiler
H12 ST-21 complex 50 Human
H54 ST-21 complex 1214 Human
B07 ST-21 complex 50 Broiler
B14 ST-21 complex 50 Broiler
H34 ST-21 complex 50 Human
H35 ST-21 complex 50 Human
H08 ST-21 complex 3769 Human
H11 ST-21 complex 21 Human
H40 ST-61 complex 61 Human
H57 ST-61 complex 61 Human
W21 ST-1275 comp. 1223 Audouin's gull
W22 ST-1275 comp. 1268 Audouin's gull
W15 ST-1275 comp. 1223 Audouin's gull
H72 S 441 Human
B45 ST-354 complex 354 Broiler
H01 S 441 Human
B44 S 441 Broiler
W05 ST-1034 comp. 4001 Audouin's gull
W19 ST-1275 comp. 3629 Audouin's gull
W11 S 1261 Audouin's gull
H09 ST-206 complex 572 Human
H52 ST-21 complex 21 Human

Figure S1

Strain	*cdtA*	*cdtB*	*cdtC*	*cadF*	*ciaB*	*htrA*	*hcp*	*virB11*	ST	ST-Complex	Species
H68									19	21	
B20									21	21	
B36									21	21	
B46									21	21	
H06									21	21	
H11									21	21	
H32									21	21	
H52									21	21	
H59									21	21	
B07									50	21	
B14									50	21	
B50									50	21	
H12									50	21	
H34									50	21	
H35									50	21	
H66									50	21	
H73									50	21	
B06									883	21	
B17									883	21	
H37									883	21	
H60									883	21	
H54									1214	21	
H08									3769	21	
H02									4664	21	
H61									459	42	
H58									4016	42	
B27									45	45	
B31									45	45	
H48									45	45	
W30									45	45	YLGB
W32									45	45	FP
W36									45	45	FP
W37									45	45	FP
W40									45	45	FP
W43									45	45	FP
W44									45	45	FP
B42									137	45	
B22									652	45	
W47									8512	45	FP
B09									48	48	
H38									48	48	
W29									48	48	YLGB
H71									49	49	
H51									52	52	
H05									61	61	
H40									61	61	
H57									61	61	
H65									61	61	
H70									61	61	
W33									179	179	FP
W41									220	179	FP
W34									2209	179	FP
W39									2209	179	FP
W46									2209	179	FP
W48									2209	179	FP
W49									2209	179	FP
B49									46	206	
H64									227	206	
H09									572	206	
H13									572	206	
H14									572	206	
H62									572	206	
H03									257	257	
H50									257	257	
H56									257	257	
B52									367	257	
B53									367	257	
B54									367	257	
B55									367	257	
B56									367	257	
B57									367	257	
B13									2254	257	
H67									2254	257	
H69									2254	257	
B33									267	283	
B28									5	353	
B29									5	353	
H63									353	353	
B48									356	353	
B05									400	353	
B18									400	353	
B30									400	353	
B40									400	353	
H36									400	353	
B23									354	354	
B45									354	354	
H49									354	354	
H74									354	354	
W28									354	354	YLGM
W54									354	354	CR
W55									354	354	WS
W56									354	354	WS
B37									8498	354	
H46									51	443	
H19									5799	443	
W12									3552	446	AGA
B08									464	464	
B15									464	464	
H18									464	464	
B21									305	574	
B25									305	574	
B04									607	607	
B41									607	607	
B38									904	607	
B47									904	607	
H04									904	607	
B24									1707	607	
B19									7110	607	
B51									7110	607	
W50									8513	952	CR
W51									8513	952	CR
W52									8513	952	CR
W05									4001	1034	AGD
W24									637	1275	YLGM
W27									637	1275	YLGM
W03									1223	1275	AGD
W10									1223	1275	AGD
W15									1223	1275	AGD
W18									1223	1275	AGD
W21									1223	1275	AGD
W22									1268	1275	AGD
W04									1275	1275	AGD
W09									1275	1275	AGD
W20									1275	1275	AGD
W14									1292	1275	AGA
W16									1292	1275	AGA
W26									3049	1275	YLGM
W19									3629	1275	AGD
W25									8511	1275	YLGM
B44									441		
H01									441		
H72									441		
B16									531		
H53									531		
W02									996		NS
W11									1261		AGD
W13									1343		AGA
W23									1343		AGA
B10									1710		
B26									1710		
B35									1710		
H07									1710		
B02									2331		
W17									2351		AGD
W06									4355		AGA
W07									4355		AGA
W08									4355		AGA
B39									7114		
H33									8479		
W53									8514		CR

Figure S2

Table S1. Breakpoints used in the antimicrobial susceptibility tests.

Antibiotic	Concentration (µg/disc)	Inhibition halos (mm)		
		R	I	S
**Ampicillin (AMP)***	10	≤ 13	14-16	≥ 17
**Amoxicillin/clavulanic acid (AMC)***	30	≤ 13	14-17	≥ 18
**Tetracycline (TET)****	30	< 30		≥ 30
**Imipenem (IMP)***	10	≤ 19	20-22	≥ 23
**Erythromycine (ERY)****	15	< 20		≥ 20
**Ciprofloxacin (CIP)****	5	< 26		≥ 26
**Nalidíxic acid (NAL)***	30	≤ 13	14-18	≥ 19
**Kanamycin (K)***	30	≤ 13	14-17	≥ 18
**Gentamicin (G)***	10	≤ 13	13-14	≥ 15
**Streptomycin (S)***	10	≤ 11	12-14	≥ 15
**Chloramphenicol (CHL)***	30	≤ 12	13-17	≥ 18
**Fosfomycine (FOS)***	200	≤ 12	13-15	≥ 16

*CLSI breakpoints; **:EUCAST breakpoints

Table S2. Primers used in this work

TARGET GENE	FUNCTION	PRIMERS	SEQUENCE (5'-3')	PCR product (bp)	Sequence source
cadF	Fibronectin-binding protein	cadF-F	CCAGGGATTAGACTTGGTTATC	203	81-176[a]
		cadF-R	AAATCCTCATATCCTCCACCTG		
*cdtA**	Cytolethal distending toxin subunit A	cdtA-F	CTATTACTCCTATTACCCCACC	422	81-176
		cdtA-R	AATTTGAACCGCTGTATTGCTC		
*cdtB**	Cytolethal distending toxin subunit B	cdtB-F	AGGAACTTTACCAAGAACAGCC	531	81-176
		cdtB-R	GGTGGAGTATAGGTTTGTTGTC		
*cdtC**	Cytolethal distending toxin subunit C	cdtC-F	ACTCCTACTGGAGATTTGAAAG	339	81-176
		cdtC-R	CACAGCTGAAGTTGTTGTTGGC		
htrA	Protease	htrA-F	TGTGAATCCTGCTGCTGGAAAT	471	81-176
		htrA-R	CCAAAAGGATTTCCAAGTGCAA		
ciaB	Invasion antigen B	ciaB-F	CCTTATGCGAGATTTTTCGAGA	504	81-176
		ciaB-R	ATTTGGAACGACTTGAGCTGAG		
hcp	T6SS	hcp-Fw	CAAGCGGTGCATCTACTGAA	463	108[b]
		hcp-R	TAAGCTTTGCCCTCTCTCCAAT		
virB11	pVir protein	virB11_P-fw	TCTTGTGAGTTGCCTTACCCCTTTT	494	81-176
		virB11_P-rv	CCTGCGTGTCCTGTGTTATTTACCC		
gltA	Housekeeping gene	gltA-F	GCCCAAAGCCCATCAAGCGGA	141	81-176
		gltA-R	GCGCTTTGGGGTCATGCACA		

[a]*C.jejuni* 81-176 complete genome sequence (accesion number CP000538)

[b]*C.jejuni* 108 type VI secretion gene locus, complete sequence (accesion number JX436460)

* Primers described in Martinez, I., Mateo, E., Churruca, E., Girbau, C., Alonso, R., and Fernandez Astorga, A. (2006) Detection of *cdtA*, *cdtB*, and *cdtC* genes in *Campylobacter jejuni* by multiplex PCR. *Int. J. Med. Microbiol.* **296**: 45–48.
